# *In vitro* forestomach digestion experiments give less-biased estimates of food composition in odontocetes

**DOI:** 10.1242/bio.059440

**Published:** 2022-10-26

**Authors:** Lisa Klemens, Carolin Julie Neven, Tom Bär, Uwe Krumme, Michael Dähne

**Affiliations:** ^1^ Deutsches Meeresmuseum, Katharinenberg 14-20, 18439 Stralsund; ^2^ Thünen Institute of Baltic Sea Fisheries, Alter Hafen Süd 2, 18069 Rostock

**Keywords:** Marine mammal, Food analyses, Stomach physiology, *In vitro* digestion

## Abstract

Diet composition of odontocetes is usually inferred from stomach content analyses and accounts for digestion rates derived from *in vitro* digestion experiments based on seal physiology. However, pinnipeds, being carnivores, have only one stomach compartment, while odontocetes, being cetartiodactyla, have up to four. Inappropriate extrapolation from digestion processes in simulated seal stomachs may result in biased estimates of odontocete diets. We simulated a forestomach accounting for muscle contractions and a pH=4 using *in vitro* experiments with three fish species. Whiting (*Merlangius merlangus*), black goby (*Gobius niger*) and sprat (*Sprattus sprattus*) showed highly variable exponential, sigmoid or linear digestion functions, and high digestion rates, taking between 50 and 230 min for completed digestion. Previous pinniped models (pH=2, lacking simulated muscular digestion) showed much slower and more similar digestion process. Our results suggest that present biomass intake estimates of odontocetes are biased towards bigger and fattier fish and need to be revised in general.

## INTRODUCTION

Odontocetes like harbour porpoises (*Phocoena phocoena* L.) display a purely aquatic lifestyle making it difficult to observe their foraging behavior in the wild (Pierce and Boyle, 1991). Therefore, the analysis of marine mammal food composition and biomass intake is traditionally performed via *post-mortem* stomach content analyses. Stomach content of odontocetes consists mostly of hard parts like premaxillaries or otoliths, which have distinctive features and can be used to identify prey species (Jobling and Breiby, 1986; Pierce and Boyle, 1991; Fitch and Brownell, 1968). However, during digestion, these parts lose their specific shape, or dissolve completely after a while, likely leading to biased estimates of both food composition and biomass intake (Andreasen et al., 2017). Such quantitative estimates of biomass consumption are, however, important given their role as top predators in marine ecosystems and the considerable economic and ecological significance of marine mammal­­ and fishery interactions (Pierce and Boyle, 1991).

To produce accurate estimates of relative composition and consumed biomass, it is important to understand the partial digestion of prey items in stomachs of odontoces. *In vitro* digestion experiments allow researchers to observe and understand the digestion processes under controlled conditions (Sekiguchi and Best, 1996; Jackson et al., 1987; Bigg and Fawcett 1985; Jobling and Breiby, 1986; Wijnsma et al., 1999; Christiansen et al., 2004, 2005). However, surprisingly, previous digestion experiments used for odontocetes are based on the stomach morphology and physiology of pinnipeds. Though such experiments are useful to understand degradation during digestion in seal stomachs they have also been applied to harbour porpoise digestion processes (Ross et al., 2016; Andreasen et al., 2017). Odontocetes are a part of the cetartiodactyla clade and are phylogenetically more closely related to hippotamidae and ruminants (Geisler and Uhen, 2003) than to pinnipeds. In fact, odontocetes have a completely different stomach morphology and physiology than pinnipeds. Pinnipeds and other mammalian carnivores have one stomach compartment, which includes all necessary glands to distribute gastric acid and digestion enzymes. In contrast, the stomach morphology of odontocetes displays four compartments, reflecting the phylogenetic relationship with the cetartiodactyla clade: (1) the forestomach having very strong longitudinal muscles but no glands, where the first digestion phase takes place, (2) the main stomach having glands showing strong mucosa and reduced longitudinal muscles, (3) the connecting channel, and (4) the pyloric stomach (Smith, 1972; Harrison et al., 1970).

In odontocetes like harbour porpoises, the remaining hard parts of prey species can virtually only be found in the first stomach compartment, the forestomach. Given the lack of glands, the gastric fluids, if present in the forestomach during digestion, are a result of a reflux through a roughly 1 to 1.5 cm diameter sphincter opening connecting the forestomach to the main stomach (Smith, 1972). Consequently, the hard parts found in the forestomach have most likely experienced mostly physical digestion processes and only minor chemical dissolvement.

This unique combination of an herbivore stomach in a piscivorous marine mammal shows that stomach physiology of odontocetes differs greatly from pinnipeds. Pinnipeds, like other carnivores, use chemical and physical digestions processes simultaneously in a single compartment (Olsen et al., 1996; Christiansen et al., 2004).

We developed an experimental set up that accounts more realistically for the forestomach digestion process of odontocetes and reflect all processes until the hard prey parts are completely dissolved. It also considers reflux from the main stomach to the forestomach, therefore accounting for muscular and biochemical processes.

To assess the potential bias in diet composition and biomass contribution we *in vitro* digested three common fish prey species of harbour porpoises [whiting (*Merlangius merlangus*), black goby (*Gobius niger*), sprat (*Sprattus sprattus*)] (Benke et al., 1998; Börjesson et al., 2003; Sveegaard et al., 2012; Jansen, 2013) and compare the results to previous estimates using the conventional pinniped set up and to stomach contents. Digestion processes of the adapted *in vitro* digestion experiments are compared between fish species according to size and body fatty acid profile.

## RESULTS

**Fig. 1.**
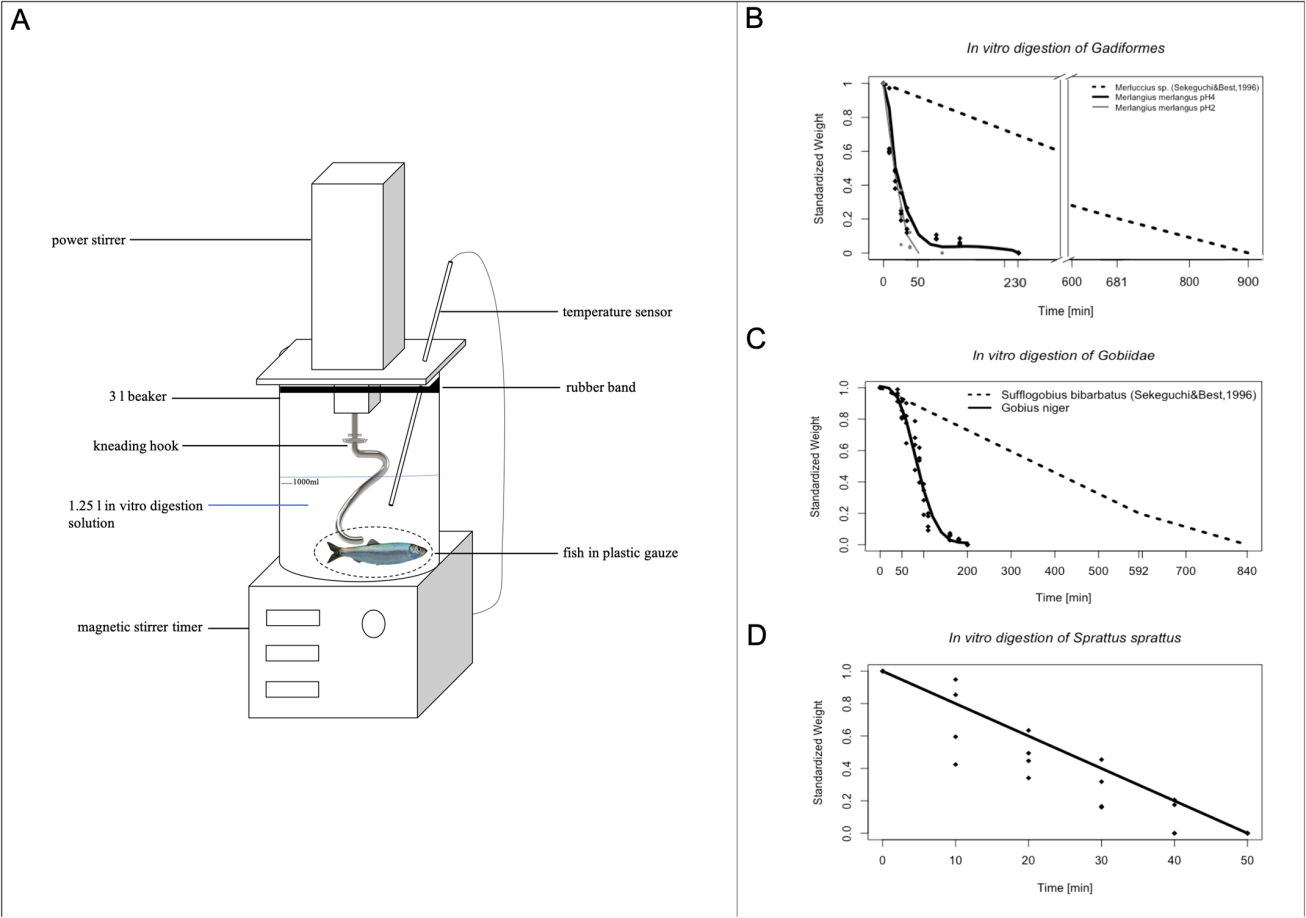
**(A) Set up of the experiment.** Gastric *in vitro* experiments were performed in a 3 *l* beaker, placed on a magnetic stirrer with a timer. The magnetic stirrer with timer kept the temperature constant, controlled with the temperature sensor. On top of the beaker a power stirring device with a kneading hook was placed. Rubber insulation was placed between the stirring device and beaker to seal the contents. The gastric fluid of harbour porpoises was simulated with a 1.25 *l* solution, following the protocol by Jackson et al. (1987) and Sekiguchi and Best (1996). For each fish species, four individual fish were wrapped in plastic gauze and digested in the solution. (B-C) *In vitro* digestion progress of four individuals of whiting (B, *Merlangius merlangus*), black goby (C, *Gobius niger*) and sprat (D, *Sprattus sprattus*) used in *in vitro* digestion experiments adapted to odontocete stomach parameters, accounting of motoric movement and pH=4 (bold line) and of five individuals of hake (B, *Merluccius sp.*) and pelagic goby (C, *Sufflogobius bibarbatus*) used in *in vitro* digestion experiments of Sekiguchi and Best (1996) without consideration of muscular digestion and pH=2 (dashed line).

**Fig. 2.**
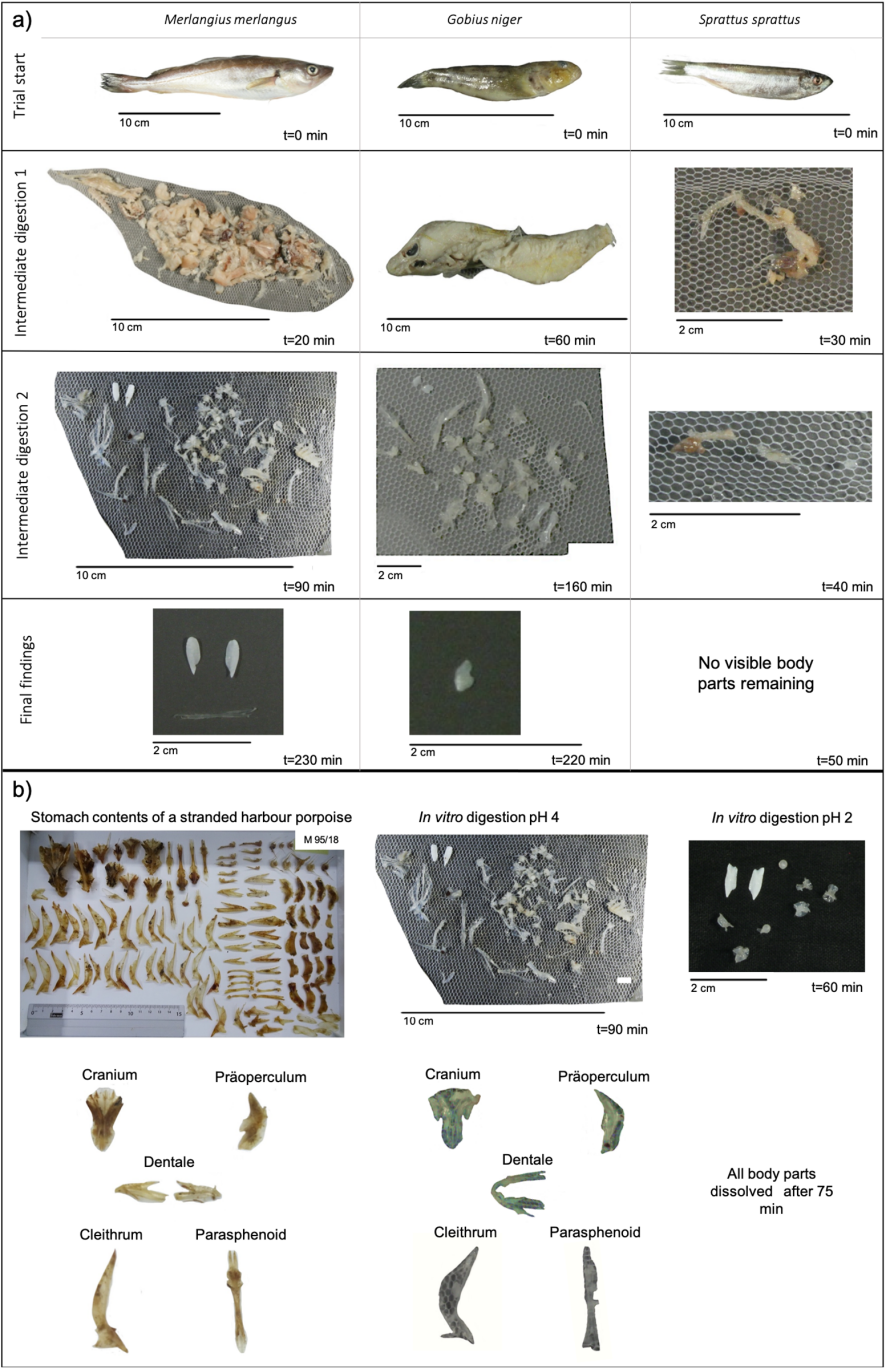
**(A) *In vitro* digestion rate of whiting (*Merlangius merlangus*), round goby (*Gobius niger*) and sprat (*Sprattus sprattus*) in an *in vitro* digestion experimental set up, which considers pH=4 and motoric movement.** (B) Comparison between hard parts of Gadidae found in the stomach of a stranded harbour porpoise (M 95/18), remaining hard parts of whiting after 90 min in the adapted *in vitro* digestion experiment with a pH value of 4 and remaining hard parts of whiting after 60 min in the adapted *in vitro* digestion experiment with a pH value of 2.

Each *in vitro* digested fish species displayed a distinctive digestion rate and the function describing the digestion process differed between the species (Figs 1B-D and 2A). Whiting showed a logarithmic digestion function (Fig. 1B), with a rapid digestion rate in the beginning, with 20% of the body weight already digested after 50 min. Each of the three whiting was completely dissolved after 230 min. The digestion of black goby resulted in a slower digestion rate and sigmoid function (Fig. 1C). Digestion rate was slow in the beginning, increased after 30 min and decreased again towards the end (after 160 min, Fig. 2A). Sprat showed a linear digestion function and a rapid digestion rate (Fig. 1D). All sprats were completely digested after 50 min.

Digestion function and rate differed greatly between the *in vitro* digestion experiments and previously performed experiments such as Sekiguchi and Best (1996). In the *in vitro* digestion experiments digestion rate was much shorter, at maximum 230 min. In addition, the digestion function was not linear in all species, the results show also logarithmic and sigmoid functions.

The remains found after digestion (using a simulation of muscular contraction and a relatively high pH=4) closely resembled the remains found in porpoise stomach analysis (Fig. 2B). Each species displayed a distinctive feature that allowed for a clear identification of prey species using nearly all bones (not only otoliths) and had fragile structures. The digestion experiments with a pH=2 dissolved all parts of fish very rapidly between 50–230 min. (Fig. 1B). The remaining parts in this experiment did not resemble hard parts found in harbour porpoise stomachs (Fig. 2B). The results indicate that digestion in odontocetes is highly influenced by motoric movement generated by the stomach musculature and to a much lower degree by acidity.

## DISCUSSION

Our study showed that the simulated digestion experiments accounting for the stomach setup of odontocetes differed greatly from previous results of *in vitro* digestion experiments using simulated pinniped stomachs by Jackson et al. (1987), Bigg and Fawcett (1985), Jobling and Breiby (1986), Wijnsma et al. (1999), Christiansen et al. (2004, 2005) and especially Sekiguchi and Best (1996) (Figs 1B-D and 2A).

### Influences of pH on digestion in odontocetes

Given the four-chambered stomach morphology of odontocetes, it is difficult to measure the real pH value of the digestion fluids in the forestomach (Mitchell et al., 2008). Digestion fluids are produced in the main stomach and flow potentially randomly as a reflux into the forestomach. Our digestion experiments and the shape and composition of the remains in dissected harbour porpoise stomachs suggest that the pH value of the digestion fluids of odontocetes is similar to the pH value of their sister taxon, the hippotamidae (pH=4.4) and to other members of the cetartiodactyla taxa like ox (pH=4.2) (Beasley et al., 2015). This more neutral pH value differs from the pH value measured in other marine predators with a single stomach compartment, such as pinnipeds which have much lower pH value of 2 (Christiansen et al., 2004).

### Stomach contractions and muscular motility

The outer epithelia of the forestomach of harbour porpoises is thick and muscular, like the ventricles of a heart. It also is surrounded by well-developed smooth musculature, just like the rumen in ruminants (Sultana et al., 2021). Rumen musculature of ruminants perform contraction cycles that increase after the ingestion of food (Kay, 1987), thereby mixing the food in the rumen.

Given the similar stomach morphology and the close taxonomic relationship between odontocetes and ruminants, it can be assumed that the forestomach of cetaceans has a similar stomach muscular motility as the rumen of ruminants. Taking the digestion processes in pinnipeds as proxy for harbour porpoise seems therefore far from adequate.

### Digestion process

The fish in our adapted in vitro experiments were digested much faster than in previous digestion experiments (e.g. Sekiguchi & Best, 1996; Jackson et al., 1987; Wijnsma et al., 1999) suggesting a strong influence of stomach muscular contractions increasing the digestion rate drastically even at a higher pH (4 instead of 2) (Fig. 1B,C). The results of the adapted *in vitro* digestion experiments show that larger fish species, like whiting, are more slowly *in vitro* digested than smaller fish, like sprat.

In the *in vitro* digestion experiments of Sekiguchi and Best (1996), similar-sized fish of comparable species took much longer to *in vitro* digest. Even for fish of lower weight and smaller size these processes lasted much longer. The pelagic gobies (*Sufflogobius bibarbatus*) used by Sekiguchi and Best (1996) were smaller than the black gobies used in our digestion experiments (Table 1), but took 240 min longer to completely digest *in vitro* (pelagic goby 840 min, black goby 240 min). Our results appear to be much closer to reality, since bycaught porpoises (dying shortly after food intake), usually have stomachs filled with already partially digested prey items while stranded animals (with an unknown time of feeding prior to death) usually only contain hard parts (observations by U.K. and M.D., Bernaldo de Quirós et al., 2018).

**Table 1.**
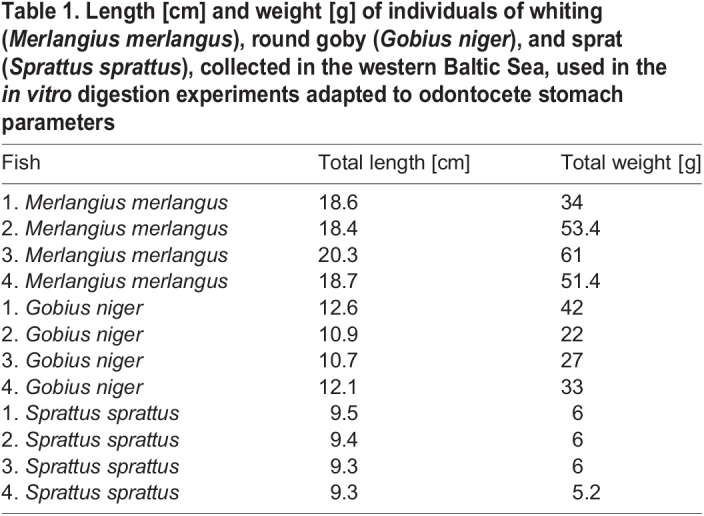
Length [cm] and weight [g] of individuals of whiting (*Merlangius merlangus*), round goby (*Gobius niger*), and sprat (*Sprattus sprattus*), collected in the western Baltic Sea, used in the *in vitro* digestion experiments adapted to odontocete stomach parameters

In our experiments, digestion rate also differed greatly between fish species (Fig. 1B-D). A possible explanation is the different fatty acid contents of the prey items. Fish species with a higher fatty acid content, in addition to pure prey size-related effects, may result in a reduced *in vitro* digestion rate. Black goby, for instance, with a high muscle C18:*n*-3 fatty acid percentage of 1.96% (Prato and Biandolino, 2011) took longest to reduce 20% of the total body weight (>100 min.) while sprat (1.54%; Gladyshev et al., 2009) and whiting (0.5%; Lie and Lambertsen, 1991) took <50 min to reduce 20% of the total body weight, resulting in different digestion rates and functions. Additional influences may be introduced by the differences in scales, skin features and outer mucous membranes. However, such differences were systematically accounted for in our experiments.

In contrast to our findings, the results of the *in vitro* digestion experiments of Sekiguchi and Best (1996) showed a linear digestion function for hake (*Merluccius sp.*) and pelagic goby (*Sufflogobius bibarbatus vb*.) (Fig. 1B-D).

Owing to the differences in digestion rate and differing digestion processes found between species together with the disparate morphology and physiology of odontocetes and pinnipeds, *in vitro* digestion experiments targeted to simulate digestion processes in odontocetes, forestomachs should therefore rigorously consider the ruminant-like stomach morphology and physiology of odontocetes accounting for stomach muscular motility and realistic pH values.

We also noticed that it is crucial to use whole fish to understand the exact digestion process, and unlike Jobling and Breiby (1986), Wijnsma et al. (1999) and Christiansen et al. (2004, 2005), who only *in vitro* digested specific skeletal parts. Using whole fish also resembles the actual feeding behavior of for e.g. harbour porpoises which intake the prey whole (Fitch and Brownell, 1968; Pierce and Boyle, 1991).

A further observation made during our experiments was that the digestion function and rate was different for each part of a fish and can influence the digestion rate of other parts. The head of sprats including the otoliths dissolved very quickly in our experiments, while goby otolith lasted for much longer and were highly protected by surrounding flesh and skin for a prolonged time period. The vertebrae of sprat on the other hand were found for a much longer period than the otoliths and can also be used for species identification (Fig. 2A). Thus, calculation of odontocete biomass consumption of sprat using otoliths counts only seems not to be adequate. Reliable estimates should account for vertebrae counts.

To allow for more realistic simulations of muscular digestion effects, histological analyses and analyses of the muscle contraction force of odontocetes are needed.

### Conclusion

Our results indicate that the digestion processes of pinnipeds and odontocetes differ significantly. Therefore *in vitro* digestion experiments based on pinniped morphology and physiology cannot be used as a proxy for odontocetes (Ross et al. 2016, Andreasen et al., 2017). The results of our experiments suggest that the digestion process of odontocetes is highly influenced by stomach muscular activity and that a higher, nearly neutral pH is more likely to reflect the *in-situ* conditions in the odontocete forestomach.

Consequently, previous estimates using the pinniped model for odontocetes are inappropriate and need revision using realistic model assumptions similar those considered in the presented study. The results also indicate that the present estimates of prey composition and biomass consumption are biased leading to an underrepresentation of and smaller fish species, like sprat, and an overestimation of larger fish, like cod (*Gadus morhua*) or whiting (*Merlangius merlangus*).

Therefore, biomass consumption and prey species composition estimates based on the pinneped model of digestion are inadequate for odontocetes.

## MATERIALS AND METHODS

*In vitro* digestion experiments help us to comprehend the digestion function and rate of prey species and therefore enables a more accurate diet and biomass composition of odontocetes. The setup of the *in vitro* digestion experiments were adapted to the morphology and physiology of odontocete stomachs.

### Set up

The experimental setup simulated the digestion conditions of the first compartment of an odontocete stomach, the forestomach (Fig. 1A). Any digestion effects on biomass consumption estimation should therefore be greatest in this compartment.

The *in vitro* digestion set up consisted of a 3 *l* glass beaker, placed on a magnetic stirrer with a timer (Steinberger Systems, SBS-MR-1600/1T) to heat the *in vitro* digestion solution to a stable temperature of 37°C, which corresponds to the temperature of skeletal musculature in harbour porpoises (Schulze, 1996). To simulate and standardise the mechanic movements inside the forestomach during digestion, a kneading hook (Bosch, Item Nr. 080060) and a power stirrer (Bosch, GSB 20-2RE, 430 W, 220 rem/min) were used. The kneading movements of the hook simulated the mixing of the ingested fish and the digestion fluids, caused by the pulse-like contraction of the surrounding stomach musculature like in cattle (Deziuk and McCauley, 1965). The power stirrer was used to move the hook with a constant force of 2 N. The force generated by the power stirrer is similar to the force generated by the smooth stomach musculature in pigs (1.14 N; Tomalka et al., 2017), which also belong to the cetartiodactyla clade (Zachos, 2015) and are considered representative for harbour porpoise stomach musculature constrictions in this experiment.

### *In vitro* digestion solution

*In vitro* samples were digested in a solution consisting of 1.250 *l* distilled water, 0.5 ml HCl (37%), 0.2 g Na_2_CO_3_ (buffer) and 12.50 g pepsin (Roth, Art. Nr. KK38,3; 2000 FIP-U/g), based on the protocol of Sekiguchi and Best (1996), Jackson et al. (1987), Bigg and Fawcett (1985), Jobling and Breiby (1986), Wijnsma et al. (1999) and Christiansen et al. (2004, 2005). Pepsin was used due to its predominant role in mammalian digestion processes (Heldmaier et al., 2004).

The *in vitro* digestion solution was adjusted to an initial pH=4, similar to the gastric pH measured in other cetartiodactyla like hippotamidae (4.4) and ox (4.2) (Beasley et al., 2015). Another trial was conducted only on whiting at a pH of 2 to resemble previous *in vitro* digestion experiments conducted for seal species.

### Experimental material

All fish were collected and frozen (−20°C) during trawl surveys in the western Baltic Sea. Fish size was deliberately chosen from the available material. Sizes and weights were standardized within species (Table 1).

Three individuals of each prey species were used, whiting (*Merlangius merlangus*, Gadidae, 18-20 cm±s.d., 34-61 g±s.d.), black goby (*Gobius niger*, Gobiidae, 10-12 cm±s.d., 33-42±s.d.) and sprat (*Sprattus sprattus*, Clupidae, 9 cm±s.d., 5-6 g±s.d.). It is currently assumed that sprat and herring are underrepresented in stomach content analysis (Andreasen et al., 2017) due to the fragile structure of their hard parts. Gobiidae and Clupeidae are of similar size, but only Gobiidae were found in high abundance, while Gadidae seem to be the most important prey species by weight (Andreasen et al., 2017).

### Experimental procedure

Before the start of the experiments, the frozen fish were thawed approximately 4°C. Each defrosted fish was photographed, measured and weighted prior to the *in vitro* digestion. To ensure that remains could be assigned to the individual fish, each fish was numbered and packed in individual plastic gauze (polyproplene) with a mesh size of 2 mm.

First, 1.25 *l* of the *in vitro* digestion solution was heated up to 37°C. Then, pepsin and four whole fish of one species were added.

All four fish packages were placed into the solution and the power stirrer was put on top of the beaker. Every 10 min each fish was photographed and weighted, taking approximately 5 min (maximum 25 min) for each fish. Before the fish were returned into the 37°C solution, the pH value was checked with a pH-meter (Akozon, HT-1202 PH-Meter) and adjusted to 4 manually if necessary, by adding HCl or Na2CO3. After the flesh of the fish was dissolved, the solution was rinsed through a sieve (mesh size: 500 μm) to check for small otoliths and small hard parts which could have passed through the gauze. The *in vitro* digestion experiments were performed until the whole fish was dissolved or the last remaining parts were no longer clearly assignable to a fish species.

For whiting, two experiments were performed with the same experimental set up, where four whiting were *in vitro* digested in a solution of pH=2 and four whiting were *in vitro* digested in a solution of pH=4. Data was analysed in R using linear regressions and GAM functions (Wood, 2011, 2004). Fatty acid profiles of the different species are examined against published literature.

### Stomach content analyses

To be able to compare the results of the adapted *in vitro* digestion experiments to *in vivo* digestion processes in harbour porpoises, we examined the stomach contents of 30 harbour porpoises. All of the porpoises were stranded on the cost of Mecklenburg-Vorpommern, with the exception of six animals that were bycaught in German waters of the Baltic Sea in the period 2013–2019.
